# Rgnef regulates bone mass through the activation of RhoA and Rac1

**DOI:** 10.1038/s12276-025-01631-w

**Published:** 2026-01-23

**Authors:** Jiae Lee, Gong-Rak Lee, Hye In Lee, Minjeong Kwon, Taehee Kim, Jong Ran Lee, Soo Young Lee, Woojin Jeong

**Affiliations:** 1 Department of Life Science, Ewha Womans University, Seoul, South Korea; 2 Multitasking Macrophage Research Center, Ewha Womans University, Seoul, South Korea; 3 Brain Korea 21 FOUR Program, LIFE Talent Development for Future Response, Ewha Womans University, Seoul, South Korea

**Keywords:** Bone, RHO signalling

## Abstract

Rho guanine nucleotide exchange factor (Rgnef/p190RhoGEF), a RhoA-specific guanine nucleotide exchange factor, has been implicated in cancer and amyotrophic lateral sclerosis, but little is known about its role in bone. Here we investigate the roles of Rgnef in bone metabolism using Rgnef-deficient and overexpressing mice. Compared with littermate wildtype mice, Rgnef-deficient mice had increased bone mass owing to lower osteolysis and higher osteogenesis, and Rgnef-overexpressing transgenic mice had the opposite bone phenotype. Rgnef deficiency inhibited osteoclast formation and resorptive function and promoted osteoblast differentiation and mineralization, whereas Rgnef overexpression had the reverse effect. Mechanistically, Rgnef promotes osteoclastogenesis by enhancing the activity of nuclear factor kappa B (NF-κB), mitogen-activated protein kinases and AKT through the activation of RhoA and Rac1 and attenuates osteoblastogenesis through the RhoA/Rac1-mediated NF-κB activation. Moreover, Rgnef-deficient mice were protected from bone loss caused by lipopolysaccharide-induced inflammation or ovariectomy. Thus, Rgnef is a crucial regulator of bone metabolism and could serve as a potential new target for treating bone diseases.

## Rgnef regulates bone metabolism and osteoclast activity

Bone health relies on a balance between bone breakdown by cells called osteoclasts and bone building by cells called osteoblasts. Researchers explored the role of a protein called Rgnef in bone health. They found that Rgnef affects both osteoclasts and osteoblasts by controlling proteins called RhoA and Rac1, which are involved in cell movement and structure. In their study, they used mice that either lacked Rgnef or had extra amounts of it. Mice without Rgnef had stronger bones, whereas those with extra Rgnef had weaker bones. This was because Rgnef influences how well osteoclasts break down bone and how well osteoblasts build it. Researchers used various lab techniques to study these effects, including examining bone cells under a microscope and measuring specific proteins. Findings suggest that targeting Rgnef could be a new way to treat bone diseases. This summary was initially drafted using artificial intelligence, then revised and fact-checked by the author.

## Introduction

Bone undergoes metabolic remodeling, in which bone resorption by osteoclasts is followed by bone formation by osteoblasts^1^. An imbalance in bone metabolism causes many bone diseases, including osteoporosis^2^. Thus, the control of differentiation and activity of these cells has clinical implications^3^.

Osteoclasts are multinucleated cells (MNCs) derived from hematopoietic stem cells through the cooperative action of macrophage colony-stimulating factor (MCSF) and the receptor activator of nuclear factor kappa B (NF-κB) ligand (RANKL)^4,5^. RANKL binds to its receptor RANK, which recruits TRAF6 that stimulates NF-κB and mitogen-activated protein kinase (MAPK) pathways. This signaling cascade activates nuclear factor of activated T cells, cytoplasmic 1 (NFATc1), a key transcription factor that regulates the expression of osteoclast-specific genes, including *tartrate-resistant acid phosphatase* (*Acp*), *dendritic cell-specific transmembrane protein*, *cathepsin K* and *calcitonin receptor*^5^. Osteoblasts are derived from mesenchymal stem cells (MSCs). Runx family transcription factor 2 (Runx2) is a master transcription factor of osteoblast differentiation that regulates the expression of osteogenic genes, including *alkaline phosphatase* (*Alpl*), *osteocalcin* (*Ocn*) and *Sp7*^6,7^. When attached to the bone surface, osteoclasts are polarized and reorganize the actin cytoskeleton to form the sealing zone that is a hallmark of resorbing osteoclasts^8^. Rho family GTPases are crucial regulators of actin cytoskeleton rearrangement^9^. They are activated by guanine nucleotide exchange factors (GEFs) and inhibited by GTPase activating proteins (GAPs)^10,11^. Rho GTPases and RhoGEFs are known to play a positive role in osteoclast differentiation and bone resorption^12,13^. The deletion of RhoA, Rac1 or Cdc42, the best characterized Rho GTPases, results in defective osteoclastogenesis, podosome arrangement and bone resorption^14–18^. Vav3, Farp2 and Dock5, known as Rac1-GEFs, are important for actin cytoskeleton organization and resorptive activity, and global Vav3- or Dock5-knockout (KO) mice exhibit an osteopetrotic phenotype^19–21^. Fgd6, a Cdc42-GEF, coordinates cell polarity and endosomal membrane recycling in osteoclasts^22^. Plekhg5, a RhoA-GEF, regulates cell polarity, podosome organization and bone resorption in osteoclasts^23^.

Rho guanine nucleotide exchange factor (Rgnef), also known as p190RhoGEF and ARHGEF28, is a RhoA-specific GEF that regulates focal adhesion formation and cell motility downstream of integrins^24–27^. The importance of Rgnef as a RhoGEF and RNA binding protein in cancer and amyotrophic lateral sclerosis has been reported^28–30^. However, the role of Rgnef in bone is largely unknown. Here, we demonstrate that Rgnef regulates the differentiation and function of osteoclasts and osteoblasts by modulating RhoA and Rac1 activity. Rgnef-KO and overexpressing transgenic (Tg) mice exhibited increased and decreased bone mass, respectively. In addition, Rgnef-KO mice were resistant to bone loss induced by inflammation or ovariectomy (OVX). Our findings indicate that Rgnef is an important regulator of bone metabolism and a useful novel target for treating bone diseases.

## Materials and methods

### Antibodies

Rabbit polyclonal antibodies against Rgnef were generated using a peptide (amino acids 1247–1265 of mouse Rgnef, used for immunoblotting) conjugated to keyhole limpet hemocyanin and maltose binding protein-tagged C-terminal peptide (amino acids 1516–1700, used for immunohistochemistry) as antigens (AbClon), and they were purified on a protein A Sepharose column. A rabbit polyclonal antibody against β-actin was purchased from Abcam. Rabbit polyclonal antibodies against Vav3 and Dock5 were purchased from MyBioSource and Bethyl Laboratories, respectively. Rabbit polyclonal antibodies against phosphorylated (p-) Jun N-terminal kinase (JNK), p-extracellular signal-regulated kinase (ERK), p-p38, p-AKT, p-transforming growth factor-β-activated kinase 1 (TAK1) and p-IκBα and rabbit monoclonal antibodies against p-p65 and AKT were purchased from Cell Signaling Technology. Rabbit polyclonal antibodies against JNK1, p38 and IκBα and mouse monoclonal antibodies against NFATc1, ERK2, TAK1 and p65 were purchased from Santa Cruz Biotechnology. Mouse monoclonal antibodies against Runx2 were purchased from Medical and Biological Laboratories.

### Mice

Rgnef-KO and Rgnef-Tg mice were provided by Dr. Jong Ran Lee (Ewha Womans University), and their generation was previously described^31,32^. All mice were fed sterile food and water at the animal facility of Ewha Womans University. All animal experiments were approved by the International Animal Care and Use Committee of Ewha Womans University. The genotypes of littermate wildtype (WT) and Rgnef-KO mice were identified by polymerase chain reaction (PCR) using three primers (5′-TTTGAGAACGGACTCCTG-3′, sense for both genotypes; 5′-CGTCATCATCATCATCATCAC-3′, antisense for WT and 5′-CATTCTGCACGCTTCAAAAG-3′, antisense for KO). The genotypes of littermate WT and Rgnef-Tg mice were determined by PCR using two primers (5′- GATTCAGGAAGAAGAGG-3′, sense; 5′- TGGCACACTGGAGTGACTC-3′, antisense).

### Radiographic and histologic analyses

The trabecular morphometry of the mouse femurs was measured within the area between 1 and 2 mm distal to the growth plate using SkyScan 1172 (Bruker) and Quantum GX3 (Revvity) microcomputed tomography and quantitatively evaluated. The femurs and calvariae were fixed with 4% paraformaldehyde overnight, decalcified in 0.5 M ethylenediaminetetraacetic acid for 7 (calvariae) or 14 days (femurs), embedded in low-melting paraffin, sectioned at 4-μm thicknesses and stained for TRAP with a hematoxylin counterstain. Other femurs were fixed for 2 days in 4% paraformaldehyde and embedded in methyl methacrylate, and 7-μm sections were stained with von Kossa stain. The bone formation rate was determined by double calcein labeling. The 6-week-old male mice were intraperitoneally injected with calcein green (20 mg/kg) twice with a 4-day interval and then euthanized 3 days after the second injection. Their femurs were fixed with 4% paraformaldehyde for 2 days and sectioned at 7-μm thicknesses. Histological images were obtained with a Zeiss LSM880 Airyscan confocal microscope (NFEC-2016-05-209580) (Carl Zeiss) at Ewha Fluorescence Core Imaging Center and analyzed using Image-Pro Plus 4.5 software (Media Cybernetics).

### Serum analysis

Blood was collected by cardiac puncture, and the serum levels of C-terminal telopeptide of type 1 collagen (CTX-1) and OCN were measured using a CTX-1 ELISA kit (Immunodiagnostic Systems) and mouse Gla-OCN high-sensitive ELISA kit (Takara), respectively.

### Osteoclast differentiation and bone resorption assay

Bone marrow-derived macrophages (BMMs) were isolated from the femurs of 7- to 8-week-old male mice, as previously described^33–35^. Osteoclasts were obtained by incubating the BMMs with 20 ng/ml RANKL and 35 ng/ml MCSF^36^. They were then fixed with 4% paraformaldehyde and stained for TRAP using a leukocyte acid phosphatase cytochemistry kit (MilliporeSigma). For the bone resorption assay, osteoclasts were cultured on and then removed from dentin (Immunodiagnostic Systems). The resorption pits were stained with hematoxylin and analyzed using Image-Pro Plus 4.5 software (Media Cybernetics).

### Osteoblast differentiation and mineralization

Osteoblast precursor cells were isolated from neonatal mouse calvariae, as previously described^37^, or MSCs prepared from compact bone were used as osteoblast precursors^38^. Osteoblast precursor cells were cultured in osteogenic medium (OGM): α-MEM containing 0.1 μM dexamethasone, 10 mM β-glycerophosphate and 50 μg/ml ascorbic acid. Osteoblast differentiation and bone nodule formation were analyzed using an ALP staining and activity assay and an alizarin red S (ARS) staining and colorimetric assay (MilliporeSigma) as previously described^39^.

### Adenovirus-mediated gene transduction

BMMs and calvaria pre-osteoblasts were transduced with adenoviruses expressing a constitutively active form of RhoA or Rac1 (Cell Biolabs) at multiplicity of infection of 50 for 36 h.

### Gene expression analysis

The expression of osteoclastic and osteoblastic genes was determined using quantitative real-time PCR as previously described^40^. Total RNA was purified with Trizol reagent (Thermo Fisher Scientific) and used to synthesize complementary DNA (cDNA) with M-MLV reverse transcriptase (Promega). The amplification of cDNA using SYBR Green PCR master mix (Bioline) was conducted on a StepOnePlus real-time PCR system (Applied Biosystems). The messenger RNA (mRNA) levels of individual genes were normalized to that of actin as an internal control gene. Most primers were previously described^36,41–43^. Unique primers used in this study were as follows: Dock5 (5′-TGGTGACACAGGGACAGTGG-3′ and 5′-CACCCCAACTATGCACGTGG-3′), Rgnef (5′-AAAGAGCTGCAGCAGAACAA-3′ and 5′-ATCCTTTCCACCAAGACTGG-3′) and Vav3 (5′-CCAAAGAGTCCAGCAAACCC-3′ and 5′-CAGCAAGCTGGATCTTTCCC-3′).

### Immunofluorescence

Osteoclasts cultured on glass were fixed with 4% formaldehyde; incubated with Alexa Fluor 488-phalloidin (Thermo Fisher Scientific) to stain the actin ring, 4′,6-diamidino-2-phenylindole (DAPI; Roche) to stain nuclei or a fluorescein in situ cell death detection kit (Roche) for labeling DNA breaks; and observed using a confocal microscope (Carl Zeiss). Apoptosis is presented as the percentage of terminal deoxynucleotidyl transferase dUTP nick end labeling (TUNEL)-positive cells counted in three random fields.

### Rho GTPase activity assay

Rho GTPase activity was assessed using RhoA, Rac1 and Cdc42 pulldown activation assay kits (Cytoskeleton)^44^. GTP-bound RhoA or Rac1/Cdc42 proteins were pulled down using agarose beads conjugated with glutathione *S*-transferase-tagged Rhotekin-RBD (for GTP-RhoA) or PAK-PBD (for GTP-Rac1 and GTP-Cdc41) proteins, followed by immunoblotting against RhoA, Rac1 or Cdc42 (Cytoskeleton).

### Calvarial injection of LPS and OVX

Calvarial injections of lipopolysaccharide (LPS) (MilliporeSigma) and OVX were carried out as previously described^37,40^. LPS (12.5 mg/kg body weight) in 100 μl of phosphate-buffered saline was injected into the space between the subcutaneous tissue and the periosteum of the skulls of 8-week-old male mice on day 0 and day 2. The mice were killed 5 days after the first injection, and the calvariae were subjected to histological analysis. The 8-week-old female mice were subjected to OVX or a sham operation. Those mice were killed 4 weeks after surgery, and their femurs were subjected to radiographic and histologic analyses.

### Statistical analysis

Data are presented as the mean ± standard deviation (s.d.), and statistical significance was determined using Prism software version 5.0 (GraphPad). We used the unpaired Student’s *t*-test with a two-tailed *P* value option for single comparison and one-way analysis of variance with Tukey’s multiple comparison test for multiple comparisons. *P* values less than 0.05 were considered statistically significant: ^*^P < 0.05, ^**^P < 0.01, ^***^P < 0.005, ^#^*P* < 0.001, ^##^*P* < 0.0005, ^###^*P* < 0.0001 between the indicated groups; ns, not significant.

## Results

### Rgnef-deficient mice have increased bone mass, and Rgnef-overexpressing Tg mice have decreased bone mass

**Fig. 1:**
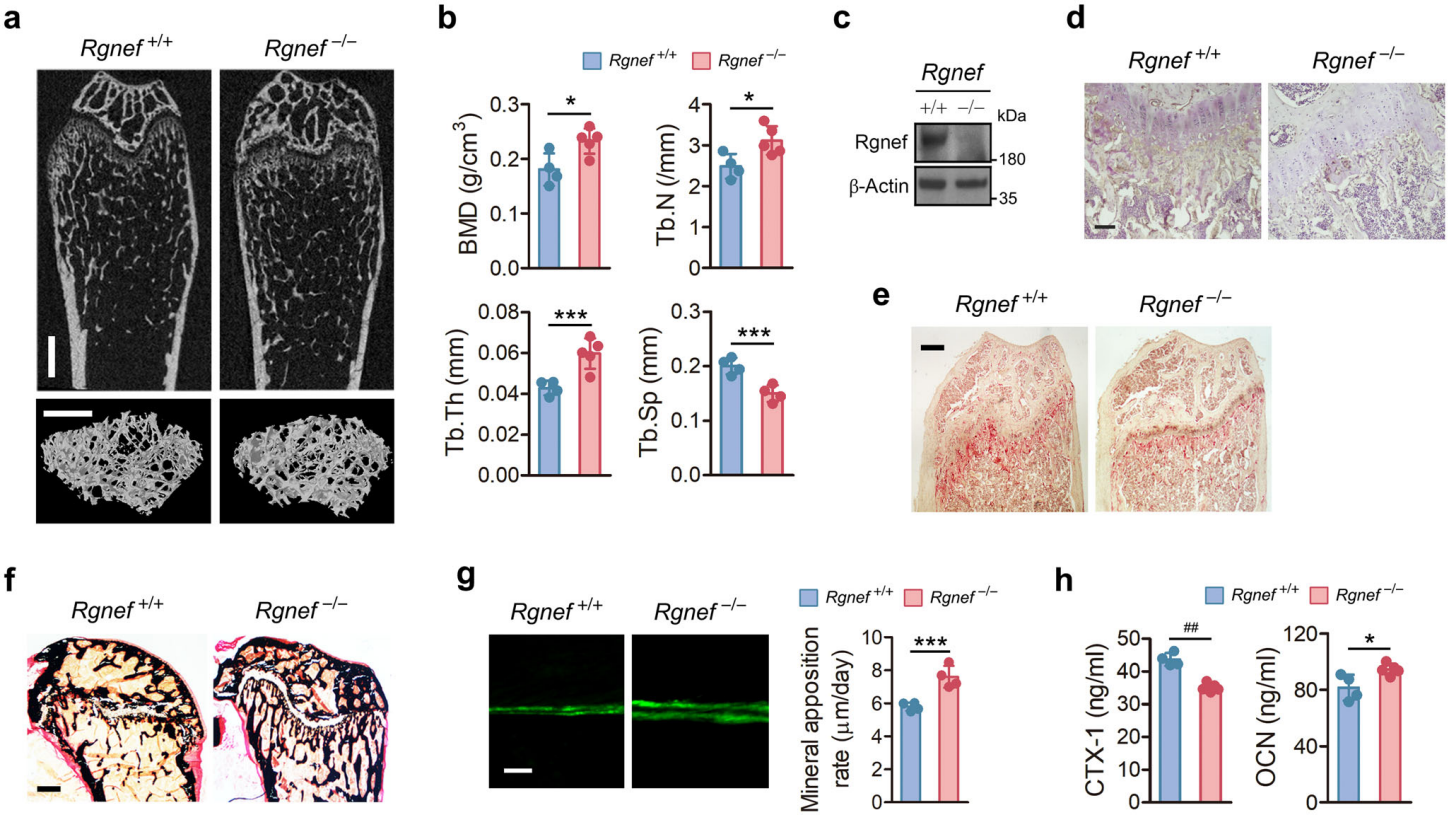
Bone phenotype of Rgnef-deficient mice in physiological conditions. **a**, Representative microcomputed tomography images of femurs from 7-week-old WT (*n* = 4) and KO mice (*n* = 5). Top, sagittal. Bottom, 3-dimensional reconstruction. Scale bar, 0.5 mm. **b**, The quantitative histomorphometry of trabecular bone. BMD, bone mineral density; Tb.N, trabecular number; Tb.Th, trabecular thickness; Tb.Sp, trabecular spacing. WT, *n* = 4; KO, *n* = 5. **c**,**d**, The determination of Rgnef protein expressed in bone by immunoblotting (**c**) and immunohistochemistry (**d**). Scale bar, 100 μm. **e**,**f**, The histological sections of femurs stained with TRAP and hematoxylin (**e**) or von Kossa/van Gieson stain (**f**). Scale bar, 100 μm. **g**, The bone formation was visualized and measured by double calcein labeling in 6-week-old WT and Rgnef-deficient mice. Scale bar, 50 μm. *n* = 4. **h**, Serum levels of CTX-1 and OCN were measured by ELISA. *n* = 4. All data are represented as the mean ± s.d. ^*^P < 0.05, ^***^P < 0.005, ^##^*P* < 0.0005. Unpaired two-tailed Student’s *t*-test in **b**, **g** and **h**.

Rgnef is a RhoGEF that specifically activates RhoA^24^. Given that Rho GTPases, including RhoA, are implicated in osteoclast formation and resorption activity^13^, we investigated whether Rgnef regulates bone metabolism using Rgnef-KO and overexpressing Tg mice. Radiographic analyses and quantitative measurements of trabecular bone showed that Rgnef-KO mice have increased bone mass (Fig. 1a, b), and Rgnef-Tg mice have decreased bone mass (Supplementary Fig. 1a,b), compared with WT mice. However, no other abnormalities were observed in Rgneg-KO and Tg mice. Rgnef protein was absent from the bones of Rgnef-KO mice, as revealed by immunoblotting (Fig. 1c) and immunohistochemistry (Fig. 1d). Histological analyses of bone sections stained for TRAP (Fig. 1e) and treated with von Kossa staining (Fig. 1f) and double casein labeling (Fig. 1g) and the analysis of serum CTX-1 and OCN levels (Fig. 1h) showed decreased osteoclastic activity and increased osteoblastic activity in Rgnef-KO mice compared with WT mice. On the contrary, the analysis of serum CTX-1 and OCN levels in Rgnef-Tg mice showed increased osteoclastic activity and decreased osteoblastic activity compared with WT mice (Supplementary Fig. 1c). These results show that Rgnef plays an essential role in the regulation of bone metabolism in vivo.

### Rgnef protein increases during osteoclast differentiation

**Fig. 2:**
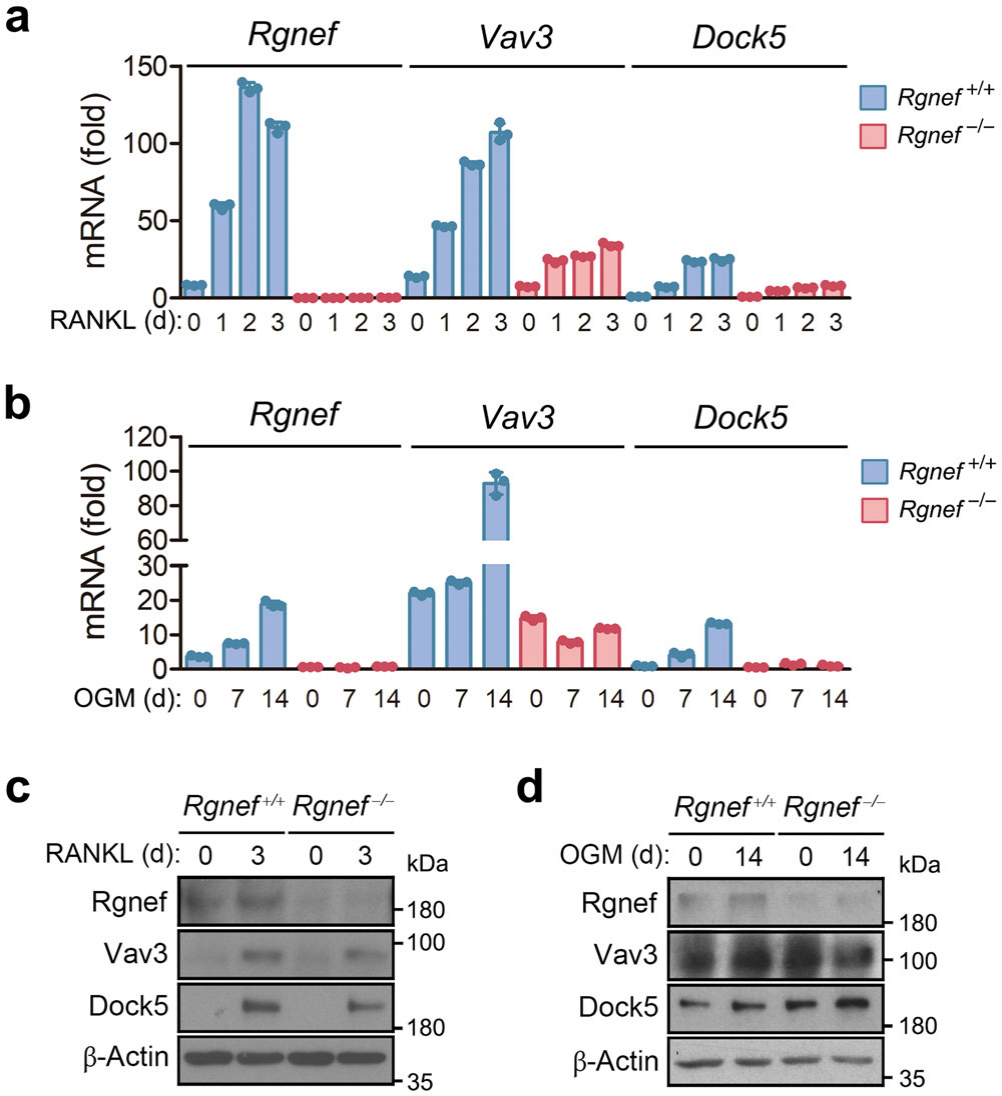
Expression of Rgnef during osteoclast and osteoblast differentiation. **a**–**d**, The BMMs and MSCs were cultured in the presence of MCSF plus RANKL (**a** and **c**) and OGM (**b** and **d**) for the indicated times, respectively. The mRNA level and protein amount of RhoGEFs was determined by real-time PCR (*n* = 3) (**a** and **b**) and immunoblotting (**c** and **d**), respectively.

Of the 42 RhoGEFs expressed in RAW264.7 cells, 7, including Dock5, Vav3 and Arhgef8, are upregulated by RANKL^45^. However, only the Dock5 and Arhgef8 are induced in RANKL-treated BMMs^19,21,45^. The Vav3 protein is observed in mature osteoblasts but not in pre-osteoblasts^19^. Therefore, we asked whether Rgnef is induced during osteoclast or osteoblast differentiation and whether Rgnef deficiency affects the expression of other RhoGEFs such as Vav3 and Dock5. The mRNA levels of Rgnef, Vav3 and Dock5 all increased during osteoclast and osteoblast differentiation, and the transcriptional induction of Vav3 and Dock5 was reduced by Rgnef deficiency (Fig. 2a, b). The protein abundances of Rgnef, Vav3 and Dock5 increased during osteoclast differentiation but not during osteoblast differentiation, and the protein expression of Vav3 and Dock5 was largely unaffected by Rgnef deficiency in both cell types (Fig. 2c, d).

### Rgnef regulates osteoclast differentiation and resorption function

**Fig. 3:**
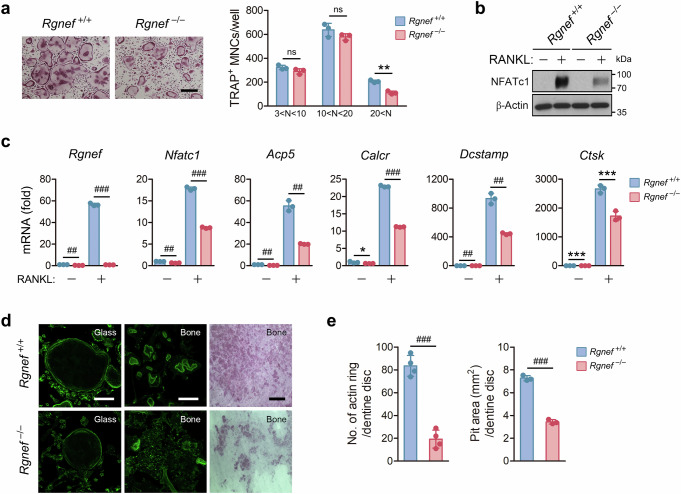
Attenuation of osteoclast formation, maturation and function by Rgnef deficiency. **a**–**c**, The BMMs were cultured in the presence of MCSF and treated with 20 ng/ml of RANKL for 4 days (**a**) or 2 days (**b** and **c**). The cells were stained with TRAP, and TRAP-positive MNCs were counted in **a**. Scale bar, 100 μm. *n* = 3. The expression of NFATc1 protein was analyzed by immunoblotting in **b** and the mRNA levels of NFATc1 target genes were determined by real-time PCR in **c**. *n* = 3. **d**, The BMMs were incubated with RANKL for 5 days on glass (left) or 7 days on dentin (middle and right). Left and middle: The cells were stained with Alexa Fluor 488-phalloidin and photographed under a confocal microscope. Right: the cells on dentin were removed, and the resorption pits were visualized by hematoxylin staining. Scale bar, 100 μm. **e**, The number of actin rings (*n* = 4) and area of the resorption pits (*n* = 3) were determined from **d**. All data are represented as the mean ± s.d. ^**^P < 0.01, ^***^P < 0.005, ^##^*P* < 0.0005, ^###^*P* < 0.0001; ns, not significant. Unpaired two-tailed Student’s *t*-test in **a**, **c** and **e**.

We next examined whether Rgnef is implicated in osteoclast differentiation and function in vitro. Rgnef-deficient BMMs differentiated into TRAP-positive MNCs, but the formation of enlarged osteoclasts containing more than 20 nuclei was reduced by Rgnef deficiency (Fig. 3a). The expression of NFATc1 and its target genes was attenuated by Rgnef deletion (Fig. 3b, c). Mature osteoclasts have a sealing zone, consisting of a ring of filamentous actin, which is essential for resorption function^46,47^. When pre-osteoclasts were differentiated on glass, the actin rings of Rgnef-deficient osteoclasts were smaller and thinner than those of WT cells (Fig. 3d). This abnormality was more evident in Rgnef-deficient osteoclasts cultured on dentin, which hardly formed actin rings at all (Fig. 3d, e). As a result, Rgnef-deficient osteoclasts dug far fewer bone resorption pits than WT cells (Fig. 3d, e). On the contrary, our experiments using Rgnef-overexpressing BMMs showed that Rgnef overexpression promotes osteoclast formation, NFATc1 activation, actin ring formation and bone resorption activity (Supplementary Fig. 2). These results indicate that Rgnef plays an essential role in osteoclast differentiation and resorption function.

### Rgnef regulates osteoclast differentiation, function and survival through RhoA and Rac1 and subsequent AKT, MAPKs and NF-κB signaling pathways

**Fig. 4:**
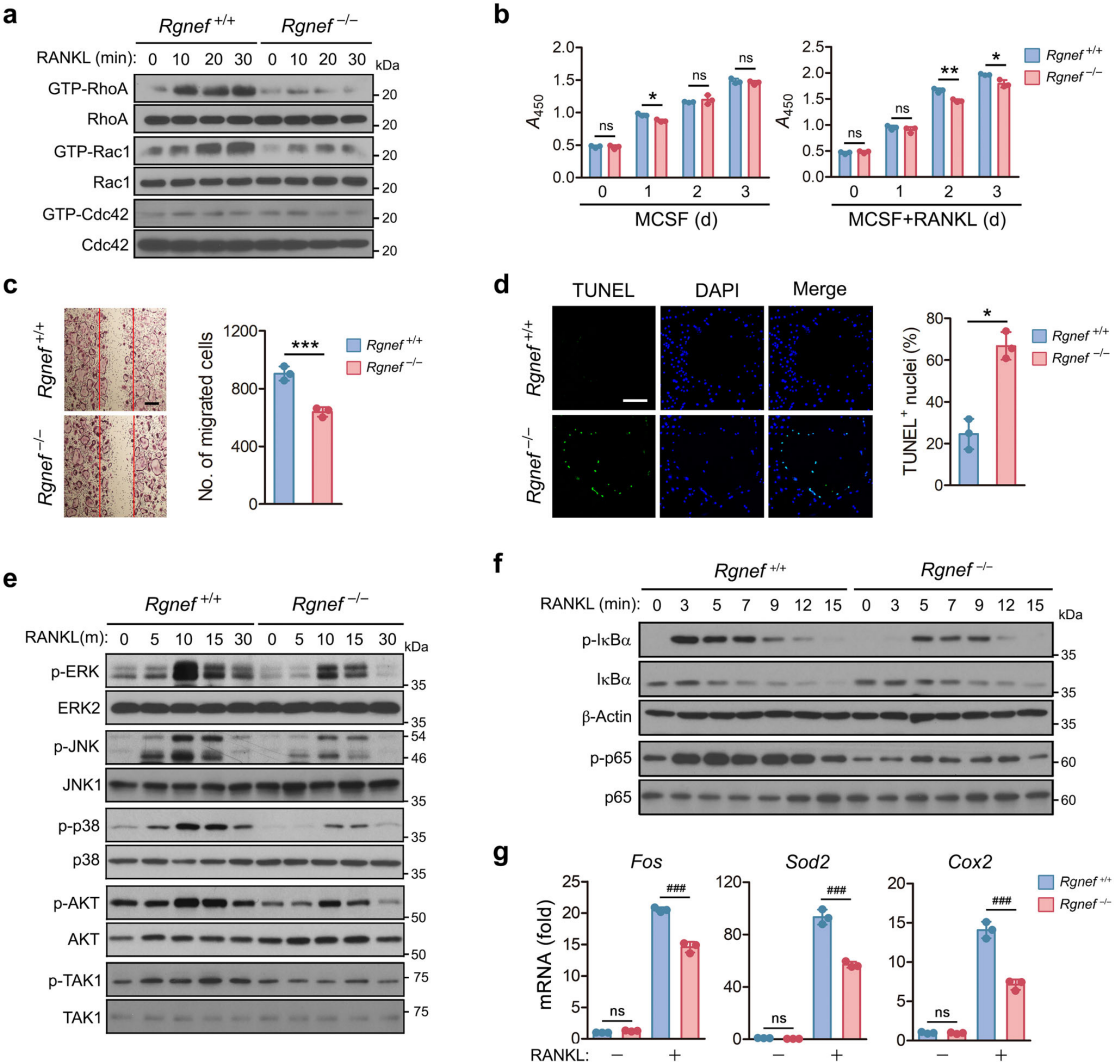
Inhibition of RhoA and Rac1 activity, survival and migration of osteoclasts by Rgnef deletion. **a**,**b**, BMMs were incubated with RANKL for the indicated times and then the activity of RhoA, Rac1 and Cdc42 (**a**), and the viability of BMMs ( **b**, left) and osteoclasts (**b**, right) were determined. *n* = 3. **c**, BMMs were cultured with RANKL for 3 days and then further incubated for 15 h after gentle scratching. Scale bars, 200 μm. The number of cells migrating into the scratched area was counted. *n* = 3. **d**, BMMs were incubated with RANKL for 4 days and then stained with TUNEL and DAPI. Scale bars, 200 μm. *n* = 3. **e**,**f**, After incubation of BMMs with RANKL for the indicated times, the phosphorylation and expression of proteins were determined by immunoblotting. **g**, After incubation of BMMs with RANKL for 12h, the transcription of NF-κB target genes was quantified by real-time PCR and is presented as fold induction. *n* = 3. All data are represented as the mean ± s.d. ^*^P < 0.05, ^**^P < 0.01, ^***^P < 0.005, ^###^*P* < 0.0001; ns, not significant. Unpaired two-tailed Student’s *t*-test in **b**–**d** and **g**.

Rho family GTPases regulate actin cytoskeleton reorganization and bone resorption of osteoclasts^9,12,13^. As Rgnef regulates actin ring formation and bone resorption, we investigated whether Rgnef is required for activation of Rho GTPases during osteoclastogenesis. The Rgnef deletion attenuated RhoA and Rac1 activation, but it had no effect on Cdc42 activation (Fig. 4a), indicating that Rgnef regulates the activation of both Rac1 and RhoA. Rac1 is involved in osteoclast apoptosis and motility^48^. Rgnef deficiency decreased the viability and motility of osteoclasts (Fig. 4b, c) and promoted apoptotic cell death (Fig. 4d). Rgnef deficiency inhibited the phosphorylation of AKT and MAPKs, including JNK, ERK and p38 (Fig. 4e). Rgnef deletion also inhibited TAK1 activation (Fig. 4e), which led to a decrease in the phosphorylation of IκBα and p65 (Fig. 4f) and the expression of NF-κB target genes (Fig. 4g).

**Fig. 5:**
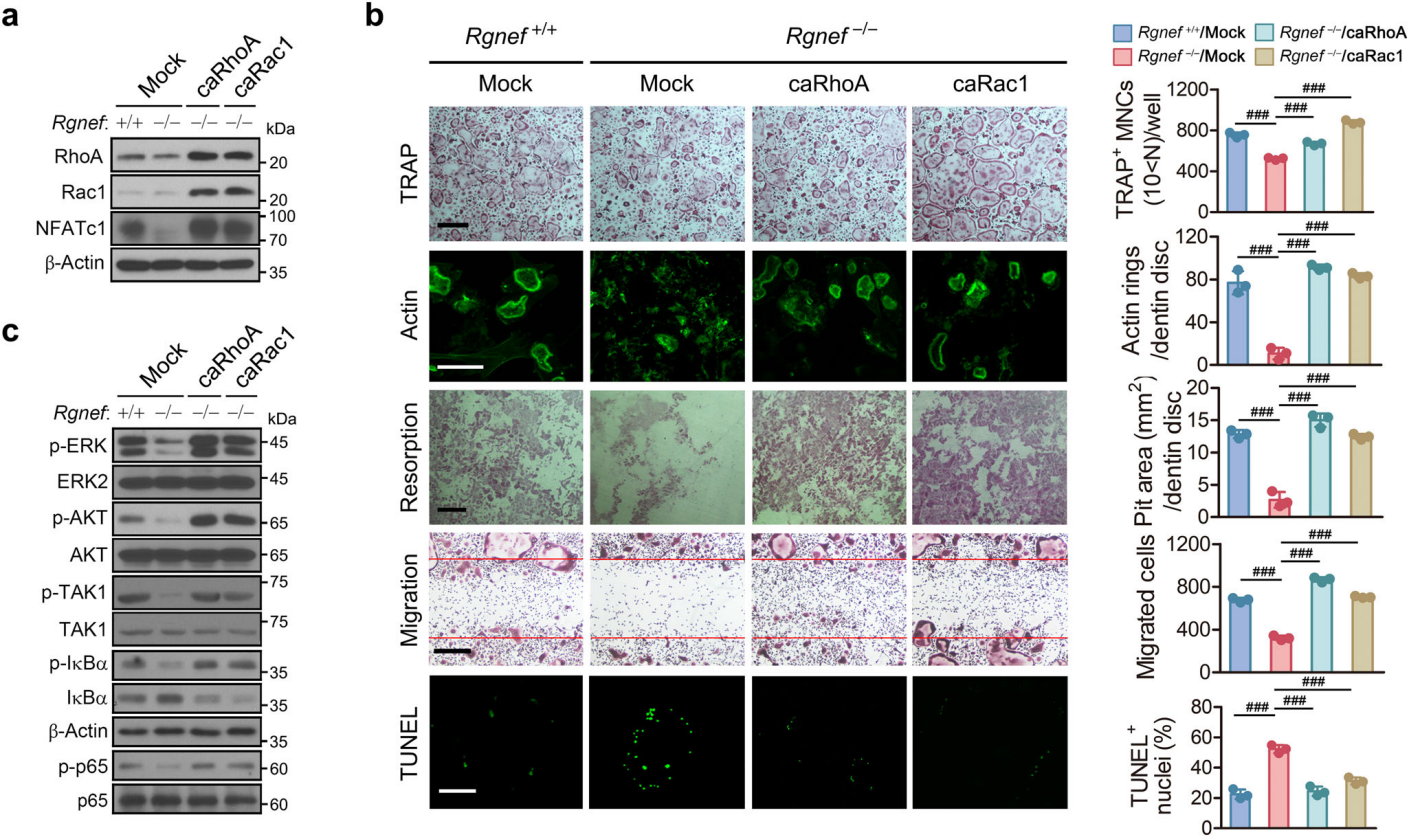
Rescue of the osteoclast differentiation, maturation, survival and function attenuated by Rgnef deficiency via the expression of RhoA or Rac1. **a**–**c**, BMMs were transduced with adenovirus expressing caRhoA or caRac1 for 36 h and then treated with RANKL (20 ng/ml) for 2 days (**a**), 3–7 days (3 days for migration; 4 days for TRAP and TUNEL staining; 5 days for actin staining; 7 days for bone resorption; **b**) and 10 min (**c**). In **a** and **c**, the phosphorylation and expression of the proteins were determined by immunoblotting. In **b**, the cells were cultured on a plate for migration and TRAP staining, on glass for TUNEL staining and on dentin to analyze actin ring formation and bone resorption. Scale bars, 200 μm in TRAP, resorption, migration and TUNEL; 100 μm in actin rings. *n* = 3. All data are represented as the mean ± s.d. ^###^*P* < 0.0001. One-way analysis of variance with Tukey’s multiple comparison test in **b**.

To confirm that Rgnef regulates osteoclast differentiation via RhoA and Rac1 activation, we transduced Rgnef-deficient BMMs with an adenovirus expressing constitutively active RhoA (caRhoA) or Rac1 (caRac1). Viral expression of RhoA or Rac1 recovered the NFATc1 expression decreased by Rgnef deletion (Fig. 5a). Remarkably, the viral transduction of RhoA induced the expression of Rac1 as well as RhoA, and Rac1 transduction showed a similar effect (Fig. 5a). Transduction with either RhoA or Rac1 restored defects in the osteoclast differentiation, actin ring formation, bone resorption, migration and survival of Rgnef-deficient cells (Fig. 5b). The expression of either RhoA or Rac1 also recovered the phosphorylation of ERK, AKT, TAK1, IκBα and p65 attenuated by Rgnef depletion (Fig. 5c). In addition, to further confirm that Rgnef regulates osteoclast differentiation through activation of the RhoA/Rac1 and NF-κB pathways, we investigated whether Rac1 or NF-κB inhibition reverses the effects of Rgnef overexpression on osteoclasts. NSC23766, a Rac1 inhibitor and BAY 11-7082, an NF-κB inhibitor, reversed the increased osteoclast differentiation induced by Rgnef overexpression (Supplementary Fig. 3a). Moreover, both Rac1 and NF-κB inhibitors reversed the effects of Rgnef overexpression on NFATc1 expression and NF-κB activation, and the Rac1 inhibitor reversed the increased AKT and ERK activation induced by Rgnef overexpression (Supplementary Fig. 3b–d). Collectively, these results suggest that Rgnef regulates osteoclast differentiation, function and survival via the activation of both RhoA and Rac1 and subsequent signaling pathways, including AKT, MAPKs and NF-κB.

### Rgnef regulates osteoblast differentiation and bone formation

**Fig. 6:**
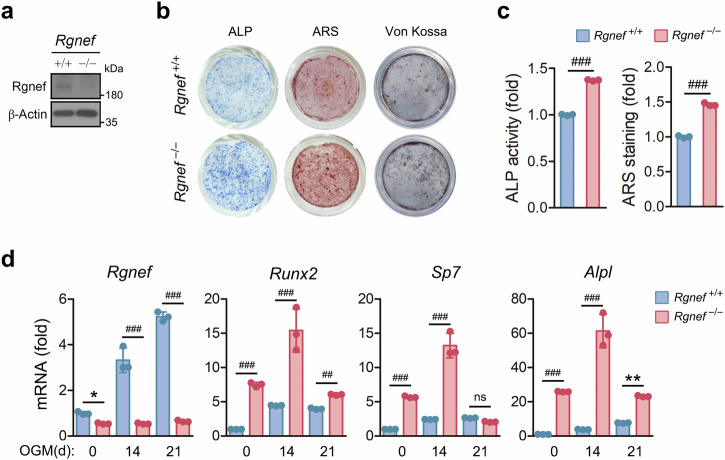
Promotion of osteoblast differentiation and function by Rgnef deletion. **a**, The expression of Rgnef protein in MSCs. **b**, The cells were fixed in 4% paraformaldehyde and stained for ALP with ARS or von Kossa stain. **c**, Cell lysates were subjected to an ALP activity assay. The ARS bound to the cells was dissolved, and the released ARS was measured. *n* = 3. **d**, Individual gene transcriptions were quantified by real-time PCR and are presented as fold induction. *n* = 3. MSCs were cultured in OGM for 14 days (**b** and **c**, ALP), 21 days (**b** and **c**, ARS), 24 days (**b**, von Kossa) or the indicated times (**d**). All data are represented as the mean ± s.d. ^*^P < 0.05, ^**^P < 0.01, ^##^*P* < 0.0005, ^###^*P* < 0.0001; ns, not significant. Unpaired two-tailed Student’s *t*-test in **c** and **d**.

Von Kossa staining, double casein labeling and serum level of OCN suggest that Rgnef inhibits osteogenic activity (Fig. 1f–h and Supplementary Fig. 1c). Therefore, we asked whether Rgnef regulates osteoblast differentiation and bone formation in vitro. ALP, ARS and von Kossa staining of cells cultured in OGM showed that Rgnef deletion promotes osteoblast differentiation and bone nodule formation (Fig. 6a–c), whereas Rgnef overexpression inhibits those processes (Supplementary Fig. 4a–c). In addition, Rgnef deficiency promoted the transcription of osteogenic genes, including *Runx2*, *Sp7* and *Alpl* (Fig. 6d), whereas Rgnef overexpression reduced it (Supplementary Fig. 4d). These results indicate that Rgnef promotes osteoblast differentiation and bone formation.

### Rgnef regulates osteoblast differentiation and function through the activation of RhoA/Rac1–NF-κB pathway

**Fig. 7:**
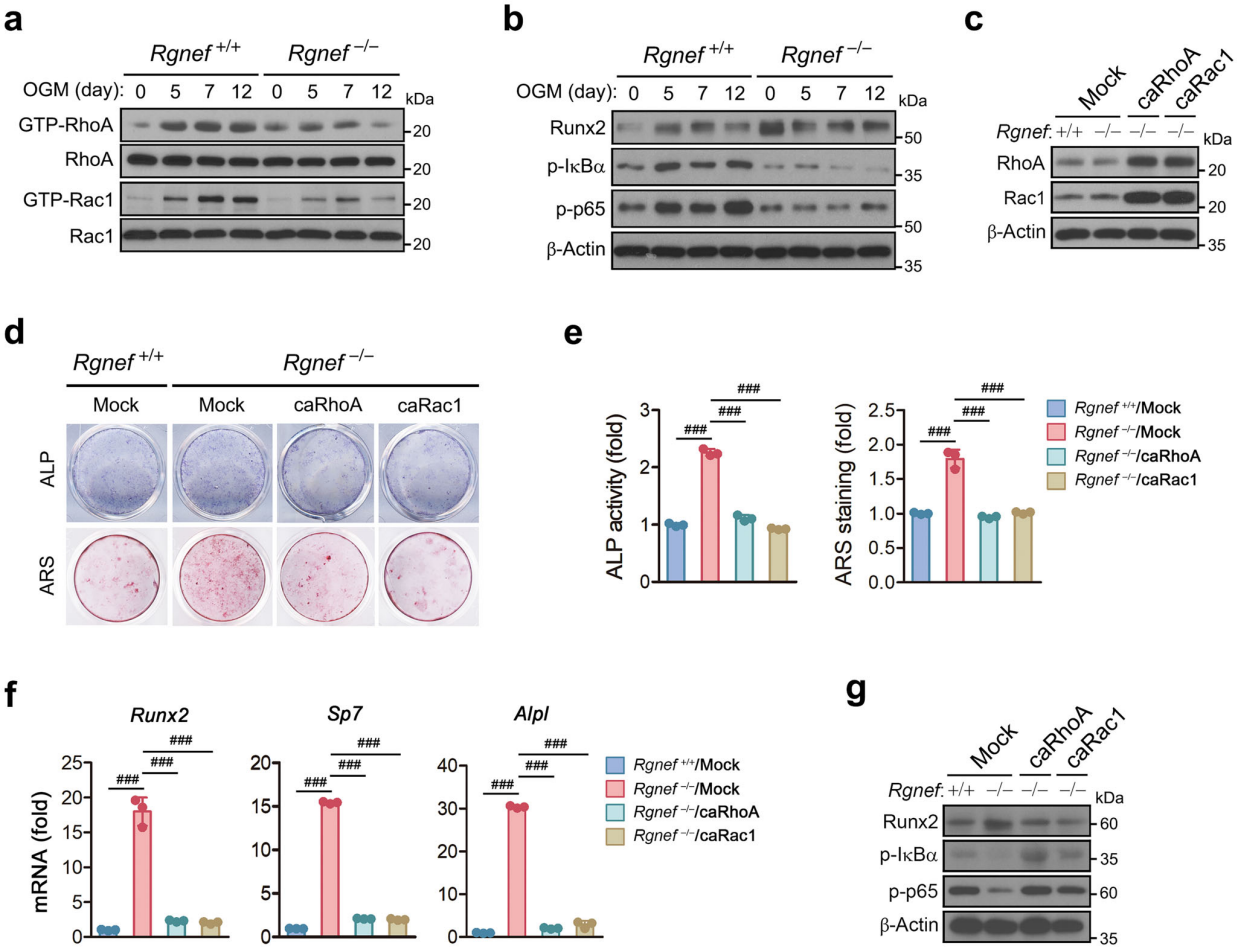
Reversal of the enhanced osteoblast differentiation and decreased RhoA/Rac1–NF-κB activation by Rgnef deletion via RhoA or Rac1 expression. **a**, The activity of RhoA and Rac1 was determined by measuring the amount of GTP-bound RhoA and Rac1, respectively. **b**,**c**,**g**, The phosphorylation and expression of the proteins were determined by immunoblotting. **d**, The cells were fixed and stained for ALP and with ARS. **e**, ALP activity and the amount of ARS bound to the cells were assessed. *n* = 3. **f**, The transcription of osteoblastic genes was quantified by real-time PCR and is presented as fold induction. *n* = 3. The osteoblast precursor cells were cultured in OGM for the indicated times in **a** and **b** or for 4 days in **c**, 12 days in **d** and **e** (ALP), 16 days in **d** and **e** (ARS), 7 days in **f**, 10 min in **g** (p-IκBα and p-p65) or 14 days in **g** (Runx2) after transduction with adenovirus expressing caRhoA or caRac1 for 36 h in **c**–**g**. All data are represented as the mean ± s.d. ^###^*P* < 0.0001. One-way analysis of variance with Tukey’s multiple comparison test in **e** and **f**.

Rho-associated protein kinase and Rac1 negatively regulate osteoblast differentiation^49–51^. Therefore, we investigated whether Rgnef regulates RhoA and Rac1 activity during osteoblast differentiation. RhoA and Rac1 were activated during osteoblast differentiation, and their activations were inhibited by Rgnef deficiency (Fig. 7a). RhoA and Rac1 are linked to the activation of NF-κB, which is known to play a negative role in osteoblast differentiation^52,53^. Rgnef deletion suppressed the phosphorylation of IκBα and p65 along with increased Runx2 expression during osteoblast differentiation (Fig. 7b).

To confirm that Rgnef regulates osteoblast differentiation and function through RhoA and Rac1 activation, we investigated the effect of ectopically expressed caRhoA or caRac1 on osteoblast differentiation and function of Rgnef-deficient osteoblast precursors. The viral expression of caRhoA or caRac1 in osteoblast precursors induced their own and the other’s expression (Fig. 7c). Either caRhoA or caRac1 expression attenuated the osteoblast differentiation and bone nodule formation augmented by Rgnef deficiency, as revealed by ALP and ARS staining, an ALP activity assay and quantitative measurement of osteogenic gene expression (Fig. 7d–f). In addition, caRhoA or caRac1 expression reversed the decreased phosphorylation of IκBα and p65 and increased Runx2 expression by Rgnef deletion (Fig. 7g). Moreover, both Rac1 and NF-κB inhibitors reversed the effects of Rgnef overexpression on osteoblast differentiation and nodule formation, NF-κB activation and Runx2 expression (Supplementary Fig. 5). Taken together, these findings indicate that Rgnef inhibits osteoblast differentiation and bone formation by activating RhoA and Rac1 and subsequently enhancing NF-κB activation.

### Rgnef-deficient mice are protected from bone loss induced by inflammation or OVX

**Fig. 8:**
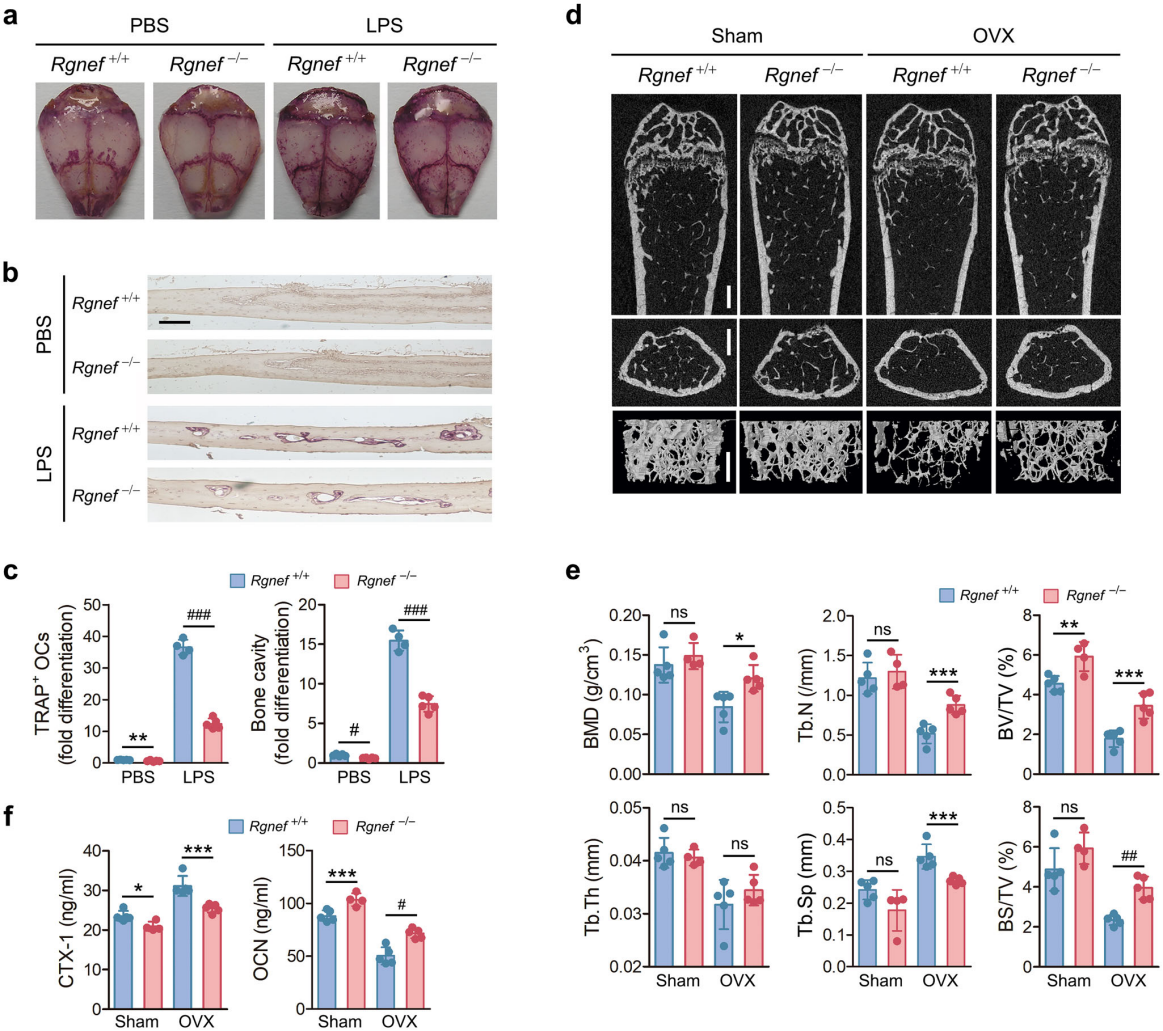
Inhibition of LPS- or OVX-induced bone destruction by Rgnef deficiency. **a**, The whole images of TRAP-stained calvariae. **b**, The section images of calvariae stained with TRAP and hematoxylin. Scale bar, 50 μm. **c**, TRAP-positive osteoclasts and bone cavities were quantified and are expressed as fold differences. In **a**–**c**, the LPS-induced bone destruction model: PBS, *n* = 5; LPS, *n* = 4 (WT) and *n* = 5 (KO). LPS-injected calvariae were fixed, stained with TRAP and decalcified. **d**, Representative microcomputed tomography images of femurs. Top, sagittal. Middle, transaxial. Bottom, three-dimensional reconstruction. Scale bar, 0.5 mm. **e**, The quantitative histomorphometry of trabecular bone: BMD, bone mineral density; Tb.N, trabecular number; BV/TV, bone volume density; Tb.Th, trabecular thickness; Tb.Sp, trabecular spacing; BS/TV, bone surface density. **f**, The serum levels of CTX-1 and OCN were measured by ELISA. In **d**–**f**, OVX model: Sham, *n* = 5 (WT) and *n* = 4 (KO); OVX, *n* = 5. All data are represented as the mean ± s.d. ^*^P < 0.05, ^**^P < 0.01, ^***^P < 0.005, ^#^*P* < 0.001, ^##^*P* < 0.0005, ^###^*P* < 0.0001; ns, not significant. Unpaired two-tailed Student’s *t*-test in **c**, **e** and **f**.

We investigated the role of Rgnef in pathological bone loss using a LPS-induced bone destruction mouse model. When LPS was injected into the calvariae of mice, the number of TRAP-positive osteoclasts and bone cavities were decreased by Rgnef deficiency (Fig. 8a–c); those were increased by Rgnef overexpression (Supplementary Fig. 6a–c). We next validated the potential of Rgnef as a therapeutic target using an OVX-induced model of postmenopausal osteoporosis. Histomorphometric analyses of femurs showed that OVX-induced bone loss is alleviated by Rgnef deficiency (Fig. 8d, e). In addition, a serum analysis showed that the extents of OVX-induced CTX-1 increase and OCN decrease are attenuated by Rgnef deletion (Fig. 8f). Similar to male mice in physiological conditions (Fig. 1f), the CTX-1 level was decreased and OCN level was increased by Rgnef deficiency in sham-operated female mice (Fig. 8f). We also pharmacologically validated the role of Rgnef in bone remodeling using NSC23766, a Rac1 inhibitor. The bone loss phenotype in Rgnef-overexpressing mice was restored by Rac1 inhibition (Supplementary Fig. 7). These results demonstrate that Rgnef depletion inhibits inflammation- and OVX-induced bone loss, and they support the potential of Rgnef as a therapeutic target for pathological bone diseases and postmenopausal osteoporosis.

## Discussion

Rho GTPases are involved in the control of multiple cellular events, including actin cytoskeleton rearrangement, cell adhesion, motility and transcriptional activation. GTPases switch between an active GTP-bound form and inactive GDP-bound form. GEFs activate GTPases by exchanging GDP with GTP. Activated GTPases return to their inactive forms when GTP is hydrolyzed to GDP via intrinsic phosphatase activity, which can be enhanced by GAPs^10,11^. The ability of osteoclasts to resorb bone depends on the formation of an actin-rich sealing zone, which is profoundly associated with actin cytoskeleton reorganization^8^. Rho GTPases and RhoGEFs promote osteoclast differentiation and bone resorption^12,13^. Thus, we hypothesized and demonstrated that Rgnef plays a negative role in bone phenotype. Rgnef-KO mice have increased bone mass (Fig. 1a, b), and Rgnef-Tg mice have decreased bone mass (Supplementary Fig. 1a, b) in physiological conditions. Moreover, Rgnef stimulates osteoclast differentiation and bone resorption in vitro, as expected (Fig. 3 and Supplementary Fig. 2), and promotes osteolysis in mice, as revealed in bone and serum analyses of Rgnef-KO and Tg mice (Fig. 1c–e, g and Supplementary Fig. 1c). Moreover, Rgnef deficiency protects against the bone loss caused by an LPS injection into the calvaria or OVX, which induces inflammation or mimics postmenopausal osteoporosis, respectively, by reducing osteoclastic differentiation and activity (Fig. 8), whereas Rgnef overexpression augments inflammation-induced bone erosion by enhancing osteoclast formation and osteolytic activity (Supplementary Fig. 6).

The effect of Rho GTPases on osteoblast differentiation was reported to be opposite in MSCs and osteoblasts^54^. A Rho GTPase activator inhibited and Rho-associated coiled-coil kinase (ROCK) inhibitors stimulated osteogenic differentiation and bone nodule formation in calvarial precursor cells^49,50^. In addition, a dominant-negative mutant of Rac1 enhanced bone morphogenetic protein 2–induced osteoblastic differentiation in C2C12 cells^51^. Meanwhile, RhoA and ROCK promote osteogenic differentiation in MSCs by regulating the commitment of stem cell fate^55^. ARHGAP18 knockdown inhibited adipogenesis and promoted the osteogenic potential of MSCs by stimulating RhoA activity^56^. It is also known that Rac1 is required in pre-osteoblasts for normal osteoblast function and bone density^57^. However, our results show that Rgnef inhibits osteoblast differentiation and bone formation in MSCs (Fig. 6 and Supplementary Fig. 4), as well as in calvarial pre-osteoblasts (Fig. 7). In addition, our bone and serum analyses of Rgnef-KO and Rgnef-Tg mice show that Rgnef inhibits osteogenesis in physiological conditions (Fig. 1f–h and Supplementary Fig. 1c), and Rgnef deletion reduces the suppression of osteogenic activity caused by OVX (Fig. 8f).

Rgnef is a RhoA-specific GEF^24–26^. However, our data show that among the well-known Rho GTPases, Rgnef regulates RhoA and Rac1 but not Cdc42 (Fig. 4a). RANKL treatment induced the activation of RhoA, Rac1 and Cdc42, but only RhoA and Rac1 activity was reduced by Rgnef deletion. In addition, the viral expression of caRhoA or caRac1 restored osteoclast differentiation, function and survival and the RANKL-induced signaling pathways suppressed by Rgnef deficiency (Fig. 5). Rgnef also regulates RhoA and Rac1 activity in osteoblasts, as revealed by the GTPase activity assay and caRhoA or caRac1 supplement experiments (Fig. 7). Taken together, these results strongly suggest that Rgnef regulates RhoA and Rac1 activity.

Mice have 76 RhoGEFs and 18 Rho GTPases^10,58^. Of the 76 RhoGEFs, 42 are expressed and 7, including Dock5, Vav3 and Arhgef8, are upregulated by RANKL in RAW264.7 cells^45^. Therefore, RhoGEFs appear to be less essential for cellular functions than Rho GTPases, and deficiency in one RhoGEF is compensated by another. However, the individual KO of Dock5 and Vav3 causes defects in osteoclast function, even though they both regulate Rac1 activity^19,21^. Rgnef deletion also suppresses osteoclast differentiation and function (Fig. 3). In our study, these three RhoGEFs, Dock5, Vav3 and Rgnef, are induced by RANKL in BMMs, though it was previously reported that Dock5 but not Vav3 is upregulated in RANKL-treated BMMs. Moreover, Rgnef deletion does not affect the RANKL-induced protein expression of Dock5 or Vav3. These observations suggest that these three RhoGEFs have distinct functions in osteoclasts that are not performed by other RhoGEFs. Compared with osteoclasts, little is known about the function and expression of RhoGEFs in osteoblasts. It was reported that Vav3 protein can be observed in mature osteoblasts but not in pre-osteoblasts^19^. In our study, the Rgnef, Dock5 and Vav3 proteins are expressed in MSCs, and their amounts do not change significantly during osteogenic differentiation.

In conclusion, our results suggest that Rgnef plays an essential role in the regulation of bone metabolism by activating both RhoA and Rac1 that increase osteolysis and decrease osteogenesis, making it a potentially useful target for the treatment of bone diseases.

## Supplementary information

Supplementary Information

## Data Availability

The data that support the findings of this study are available from the corresponding author upon reasonable request.
