# Supplementary material for: Rgnef regulates bone mass through the activation of RhoA and Rac1
Source: Exp Mol Med. 2026 Jan 23;58(1):243–53. doi: 10.1038/s12276-025-01631-w (PMC12868669; doi:10.1038/s12276-025-01631-w)
Supplement: Supplementary file 1 — Supplementary Information [file 12276_2025_1631_MOESM1_ESM.pdf]

Supplementary Information

Rgnef regulates bone mass through the activation of RhoA and Rac1

Jiae Lee[1,2,a], Gong-Rak Lee[1,2,a], Minjeong Kwon[1,2], Hye In Lee[1,2], Taehee Kim[1,2,3], Jong Ran Lee[1], Soo Young Lee[1,2,3] and Woojin Jeong[1,2,3]*

[1]Department of Life Science, Ewha Womans University, Seoul 03760, South Korea

[2]Multitasking Macrophage Research Center, Ewha Womans University, Seoul 03760, South Korea

[3]Brain Korea 21 FOUR Program. LIFE Talent Development for Future Response, Ewha Womans University, Seoul 03760, South Korea

[a] These authors contributed equally to this work.

*Corresponding author: Woojin Jeong, Ph.D., Science Building C, Room 211, 52 Ewhayeodae-gil, Seodaemun-gu, Seoul 03760, Korea. Tel: +82-2-3277-4495; Fax: +82-2-3277-3760; Email: jeongw@ewha.ac.kr

Materials

NSC23766, a Rac1 inhibitor, and BAY 11-7082, an NF-κB inhibitor, were purchased from MedChemExpress (Monmouth Junction, NJ, USA).

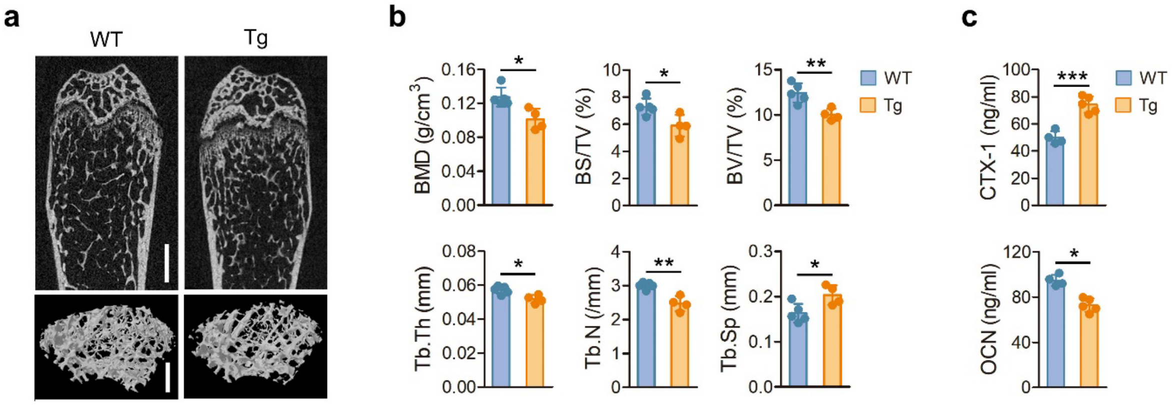

Supplementary Fig. 1. Bone phenotype of Rgnef overexpressing transgenic mice in physiological condition. (a) Representative microcomputed tomography images of femurs from 7-week-old Rgnef wild-type (WT) (*n*=5) and transgenic (Tg) mice (*n*=4): upper, sagittal; lower, three-dimensional reconstruction. Scale bar, 0.5 mm. (b) Quantitative histomorphometry of trabecular bone: BMD, bone mineral density; BS/TV, bone surface density; BV/TV, bone volume density; Tb.Th, trabecular thickness; Tb.N, trabecular number; Tb.Sp, trabecular spacing. WT, *n*=5; Tg, *n*=4. (c) Serum levels of CTX-1 and OCN in WT (*n*=4) and Tg mice (*n*=5). All data are represented as the mean ± standard deviation (SD). $^{*}P< 0.05$, $^{**}P< 0.01$, and $^{***}P< 0.005$. Unpaired two-tailed Student's *t*-test (b and c).

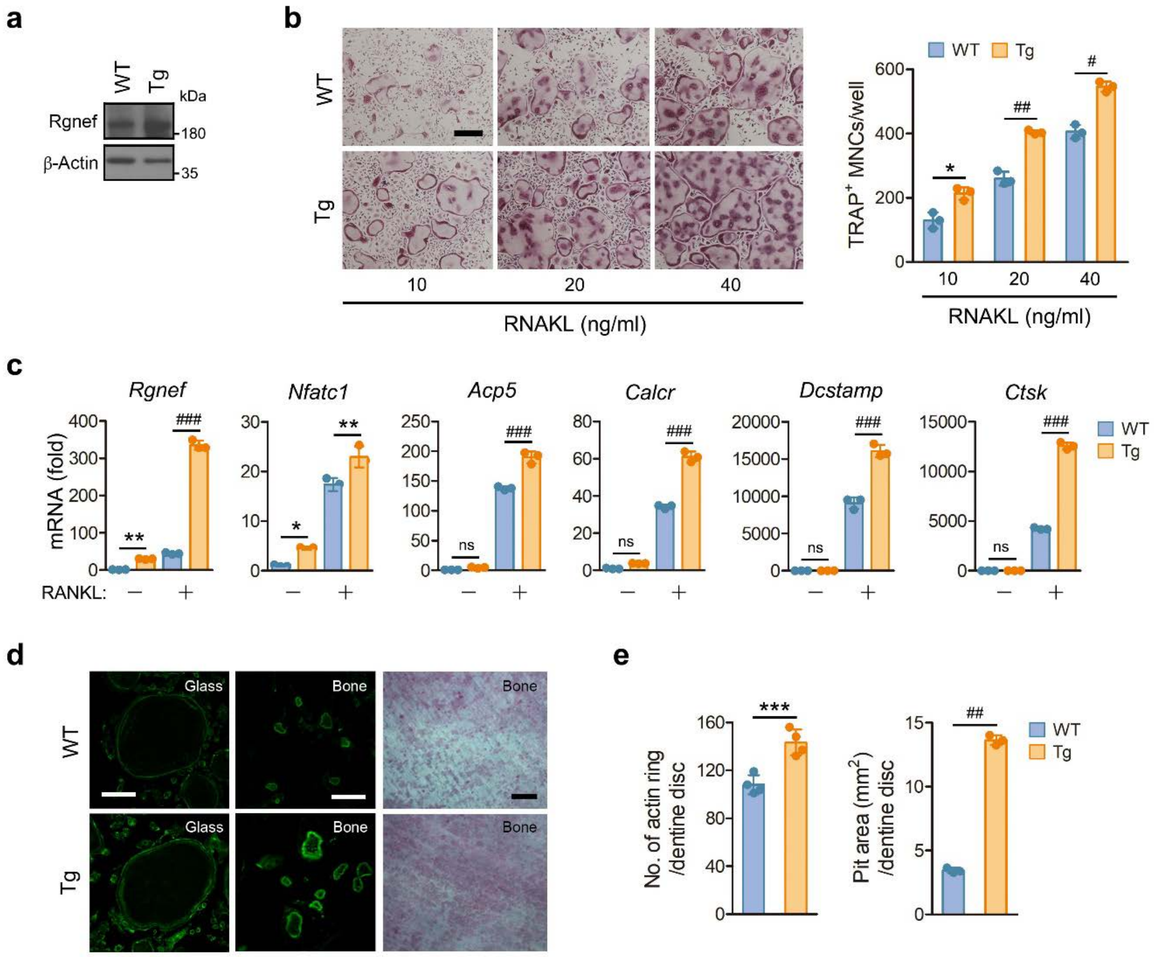

Supplementary Fig. 2. Promotion of osteoclast differentiation, actin ring formation and bone resorption by Rgnef overexpression. (a) The expression of Rgnef protein in BMMs was determined by immunoblotting. (b-e) BMMs were cultured in the presence of MCSF (35 ng/ml) and treated with the indicated concentration of RANKL for 4 days (b) or 20 ng/ml of RANKL for 2 days (c) on plate, 5 days on glass (d, *left*) or 7 days on dentin (d, *middle* and *right*). (b) The cells were fixed, subjected to TRAP staining, and visualized under a light microscope. Scale bar, 100 μm. TRAP-positive multinucleated cells (MNCs) containing more than twenty nuclei were counted. *n*=3. (c) Relative mRNA levels of *Nfatc1* and its target genes were quantified by real-time PCR and presented as fold induction. *n*=3. (d)

The cells were stained with Alexa fluor 488-phalloidin and then photographed under a confocal microscope (*left* and *middle*). The cells on dentin were removed and resorption pits were visualized by hematoxylin staining (*right*). Scale bar, 100 μm. (e) The number of actin ring (n=4) and the area of resorption pit (n=3) were determined from d. All data are represented as the mean ± SD. $^{*}P< 0.05$, $^{**}P< 0.01$, $^{***}P< 0.005$, $^{\#}P < 0.001$, $^{\#\#}P < 0.0005$, and $^{\#\#\#}P < 0.0001$; ns, not significant. Unpaired two-tailed Student's *t*-test (b, c, and e).

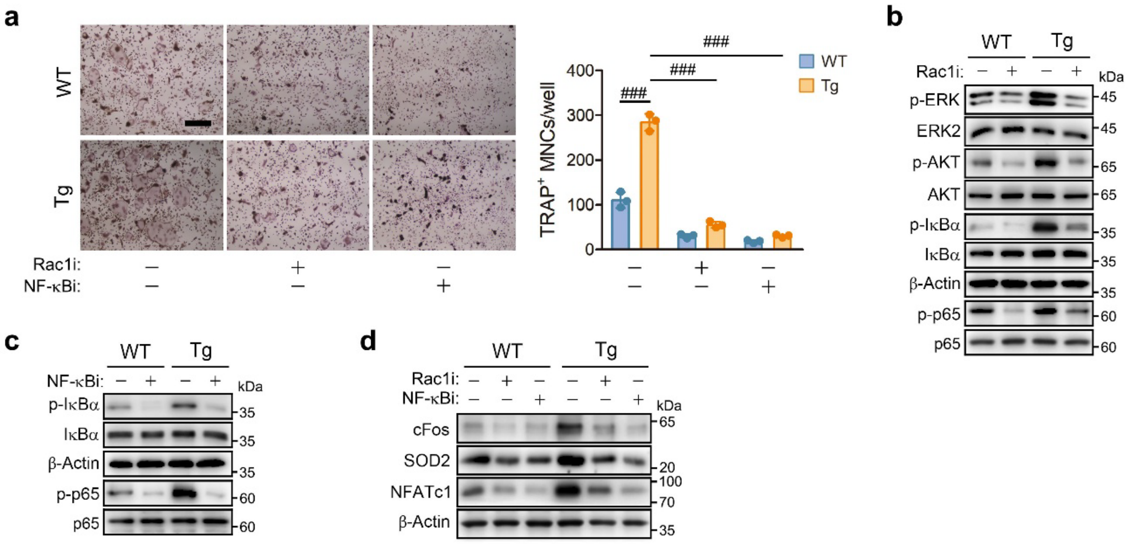

Supplementary Fig. 3. Reversal of the increased osteoclast differentiation and NF-κB activation by Rgnef overexpression through Rac1 or NF-κB inhibition. BMMs were cultured in the presence of MCSF (35 ng/ml) and treated with 20 ng/ml of RANKL for 4 days (a), 15 min (b and c), or 2 days (d) in the presence of Rac1 inhibitor (20 μM) or NF-κB inhibitor (4 μM). (a) The cells were fixed, subjected to TRAP staining, and visualized under a light microscope. Scale bar, 100 μm. TRAP-positive multinucleated cells (MNCs) containing more than twenty nuclei were counted. *n*=3. (b-d) The phosphorylation and expression of the proteins were determined by immunoblotting. All data are represented as the mean ± SD. $^{###}P < 0.0001$. One-way analysis of variance with Tukey's multiple comparison test (a).

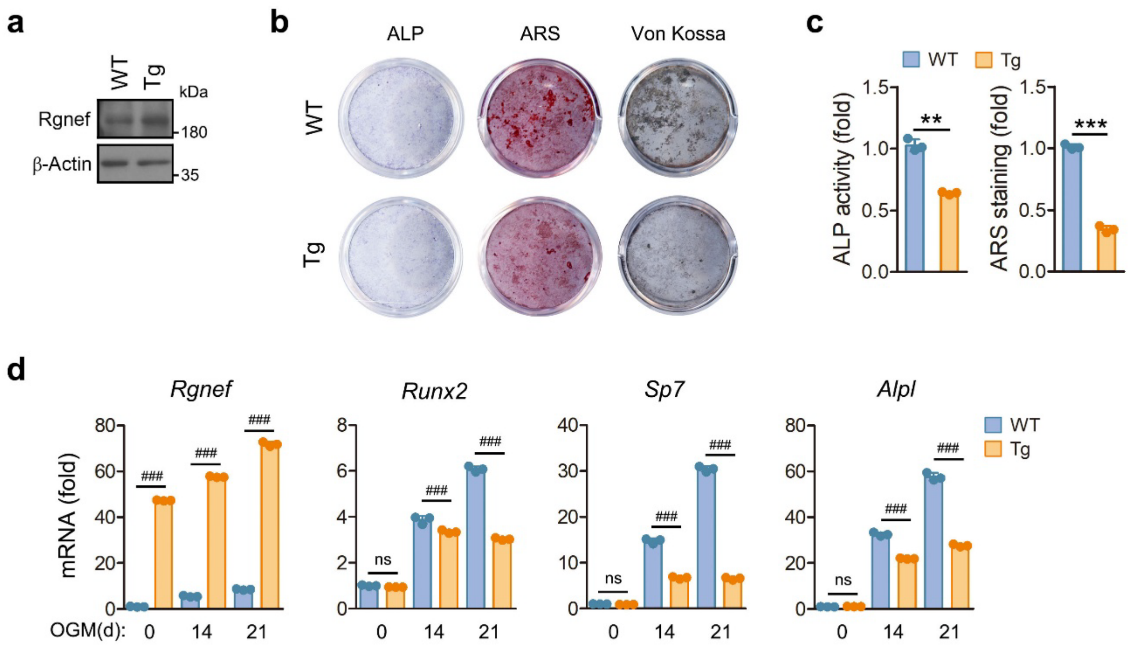

Supplementary Fig. 4. Inhibition of osteoblast differentiation and function by Rgnef overexpression. MSCs were cultured in OGM for 14 days (b and c, ALP), 21 days (b and c, ARS), 24 days (b, Von kossa), and the indicated times (d). (a) The expression of Rgnef in MSCs was determined by immunoblotting. (b) The cells were fixed in 4% paraformaldehyde and stained for ALP, with ARS or Von kossa. (c) Cell lysates were subjected to ALP activity assay, and the amount of ARS bound to cells was measured. *n*=3. (d) The transcription of osteoblastic genes was quantified by real-time PCR and presented as fold induction. *n*=3. All data are represented as the mean ± SD. $^{**}P< 0.01$, $^{***}P< 0.005$, and $^{###}P < 0.0001$; ns, not significant. Unpaired two-tailed Student’s *t*-test (c and d).

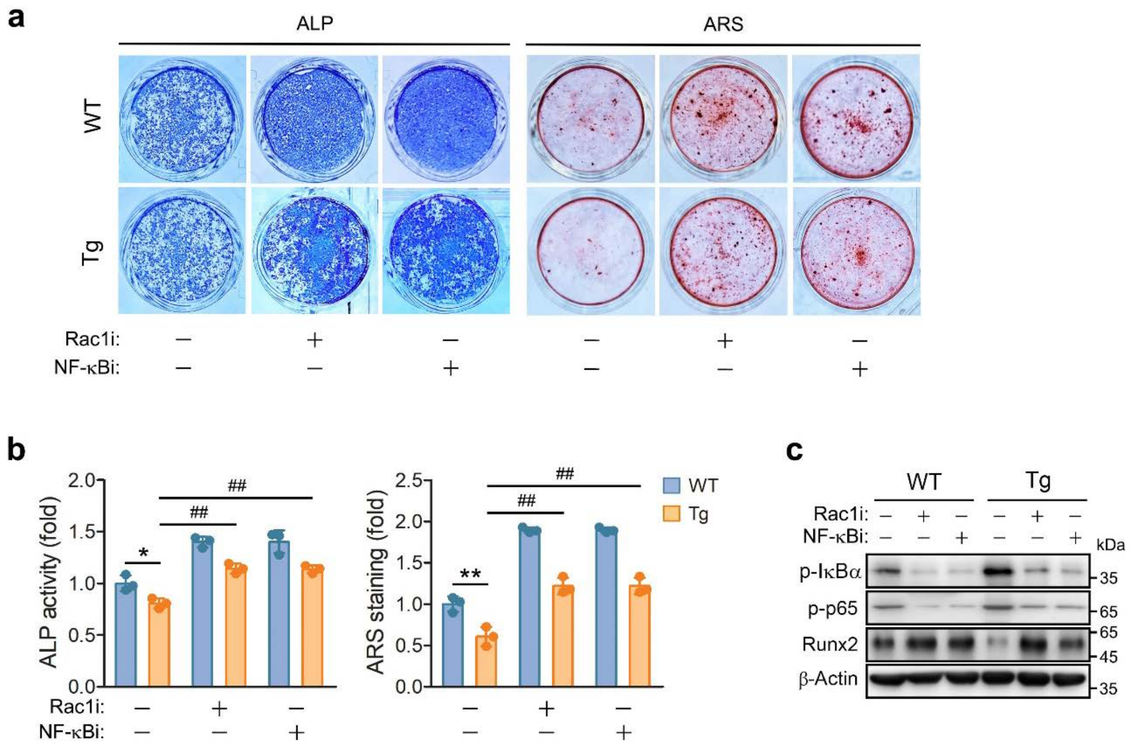

Supplementary Fig. 5. Recovery of the decreased osteoblast differentiation and increased NF-κB activation by Rgnef overexpression through Rac1 and NF-κB inhibition. Osteoblast precursor cells were cultured in OGM for 14 days (a and b, ALP), 21 days (a and b, ARS), 10 min (c, p-IκBα and p-p65), or 14 days (c, Runx2) in the presence of Rac1 inhibitor (20 μM) or NF-κB inhibitor (4 μM). (a) The cells were fixed and stained for ALP and with ARS. (b) ALP activity and the amount of ARS bound to the cells were assessed. *n*=3. (c) The phosphorylation and expression of the proteins were determined by immunoblotting. All data are represented as the mean ± SD. $^{*}P< 0.05$, $^{**}P< 0.01$, and $^{\#\#}P < 0.0005$. One-way analysis of variance with Tukey's multiple comparison test (b).

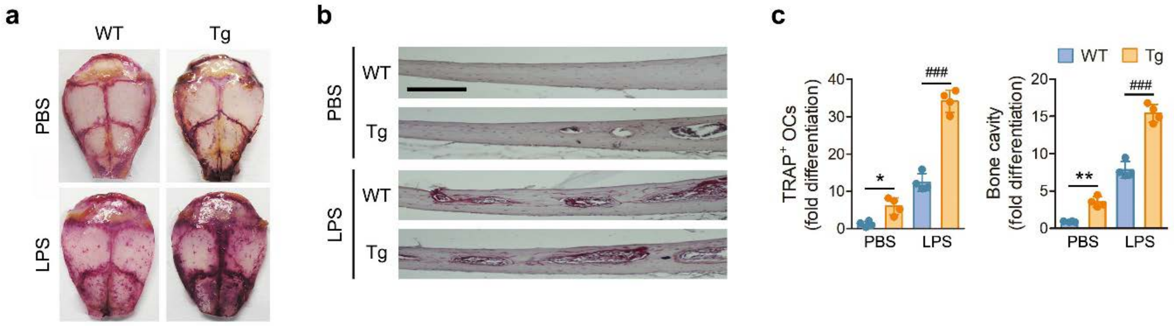

Supplementary Fig. 6. Augmentation of LPS-induced bone destruction by Rgnef overexpression. LPS-injected calvaria was fixed, stained with TRAP and decalcified. (a) Whole images of TRAP-stained calvaria. (b) Section images of calvaria stained with TRAP and hematoxylin. Scale bar: 100 μm. (c) TRAP-positive osteoclasts and bone cavity were quantified and expressed as fold difference. *n*=4. All data are represented as the mean ± SD. $^{*}P< 0.05$, $^{**}P< 0.01$, and $^{\#\#\#}P < 0.0001$. Unpaired two-tailed Student's *t*-test (c).

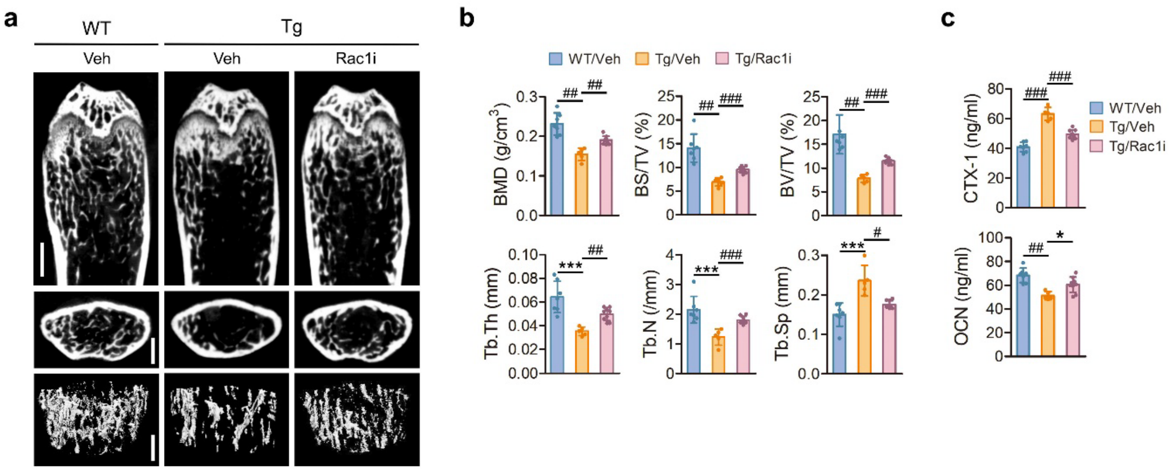

Supplementary Fig. 7. Recovery of bone mass reduction induced by Rgnef overexpression through Rac1 inhibition. (a) Representative microcomputed tomography images of femurs from 9-week-old Rgnef WT (*n*=7) and Tg mice (Veh, *n*=5; Rac1i, *n*=8): *top*, sagittal; *middle*, transaxial; *bottom*, 3-dimensional reconstruction. Rac1 inhibitor was administered intraperitoneally to mice at a dose of 2.5 mg/kg from 4 to 9 weeks of age. Scale bar, 0.5 mm. (b) Quantitative histomorphometry of trabecular bone: BMD, bone mineral density; BS/TV, bone surface density; BV/TV, bone volume density; Tb.Th, trabecular thickness; Tb.N, trabecular number; Tb.Sp, trabecular spacing. WT-Veh, *n*=7; Tg-Veh, *n*=5; Tg-Rac1i, *n*=8. (c) Serum levels of CTX-1 and OCN were measured by ELISA. WT-Veh, *n*=7; Tg-Veh, *n*=5; Tg-Rac1i, *n*=8. All data are represented as the mean ± SD. $^{*}P < 0.05$, $^{***}P < 0.005$, $^{\#}P < 0.001$, $^{\#\#}P < 0.0005$, and $^{\#\#\#}P < 0.0001$. Unpaired two-tailed Student's *t*-test (b and c).
